# Detection of early cartilage deterioration associated with meniscal tear using T1ρ mapping magnetic resonance imaging

**DOI:** 10.1186/s12891-015-0487-4

**Published:** 2015-02-10

**Authors:** Hirokazu Matsubara, Ken Okazaki, Yukihisa Takayama, Kanji Osaki, Yoshio Matsuo, Hiroshi Honda, Yukihide Iwamoto

**Affiliations:** Department of Orthopedic Surgery, Graduate School of Medical Sciences, Kyushu University, 3-1-1 Maidashi, Higashi-ku, Fukuoka, 812-0054 Japan; Department of Clinical Radiology, Graduate School of Medical Sciences, Kyushu University, 3-1-1 Maidashi, Higashi-ku, Fukuoka, 812-0054 Japan

**Keywords:** Cartilage degeneration, Meniscus, Osteoarthritis, Magnet resonance imaging (MRI), T1ρ MRI

## Abstract

**Background:**

In patients with degenerative meniscal tears, subclinical cartilage degeneration may be present even if gross morphological changes are not evident. The aim of this study was to detect occult cartilage degeneration using T1ρ MRI mapping in patients with meniscal tears without obvious radiographic osteoarthritis (OA).

**Methods:**

A total of 22 subjects with degenerative meniscal tears in the early stages of osteoarthritis [Kellgren-Lawrence (KL) grade of 0–2] and 19 healthy subjects as the control group were examined. The femoral condyle was divided into four 30° wedges (−30°–0° anteriorly, 0°–30°, 30°–60° and 60°–90° posteriorly), and each area of cartilage was further divided into superficial and deep layers of equal thickness. The tibial side was divided into anterior and posterior areas with superficial and deep layers in each. The mean T1ρ values (ms) in each area were calculated.

**Results:**

On the femoral side, T1ρ values of the superficial and deep regions (−30°–0°, 0°–30° and 30°–60°) in the meniscal tear group were significantly higher than those in the control group [superficial (−30°–0°): 49.0 ± 4.0 (meniscal tear group) vs 45.1 ± 2.1 (control group), deep (−30°–0°): 45.2 ± 3.3 vs 39.5 ± 5.0, superficial (0°–30°): 54.5 ± 5.3 vs 47.4 ± 5.7, deep (0°–30°): 46.8 ± 4.0 vs 40.7 ± 6.3, superficial (30°–60°): 50.5 ± 3.1 vs 47.1 ± 5.7]. On the tibial side, the meniscal tear group had significantly higher T1ρ values superficially in both anterior and posterior regions compared with the control group [superficial (anterior): 52.0 ± 4.3 vs 46.7 ± 5.4, superficial (posterior): 53.1 ± 5.1 vs 46.0 ± 4.9]. Moreover, these significant differences were observed when comparing patients in the meniscal tear group with KL grades of 0 or 1 and the control group.

**Conclusions:**

Our study suggested that early biochemical changes in cartilage associated with degenerative meniscal tears occur first in the superficial zones in areas of contact during slight flexion. Characterising the early relationship between cartilage degeneration and degenerative meniscal tears using T1ρ MRI mapping may be of clinical benefit and provide further evidence linking meniscal injury to OA.

## Background

Despite the high prevalence of meniscal tears, the role of meniscal injury in the pathogenesis of articular cartilage degeneration is poorly understood [1]. The menisci play an important role in knee joint stability, joint lubrication and shock absorption and help to maintain the integrity of the articular cartilage [2,3]. A number of studies have shown an association between knee osteoarthritis (OA) and meniscal damage [4-8]. These findings support the theory that disruption and excision of the meniscus contribute to cartilage degeneration or progression of OA. We have encountered several cases in which hydrarthrosis with subsequent progressive cartilage degeneration occurred after meniscectomy, although obvious morphological changes had not been detected during preoperative evaluation using radiography and conventional magnetic resonance imaging (MRI) or during intra-operative arthroscopy. We hypothesise that subclinical cartilage degeneration occurs after the occurrence of a degenerative meniscal tear, even if gross morphological changes are not evident in the cartilage. Meniscectomy is commonly performed on a patient with knee joint symptoms despite being associated with a mild OA such as that of Kellgren and Lawrence grade 2. Therefore, it would be a clinical benefit if the surgeon could preoperatively evaluate occult cartilage degeneration in patients with a meniscal tear. In addition, characterising the early relationship between cartilage degeneration and meniscal damage may provide further evidence linking meniscal injury to OA.

Early stages of OA are primarily associated with loss of proteoglycans (PG), changes in the water content and minor structural changes in collagen [9]. MRI techniques have the potential to detect such biochemical changes in the composition of joint articular cartilage non-invasively [10,11]. Specifically, T1ρ mapping has been shown to be sensitive to changes in PG loss in cartilage and has attracted attention as a non-invasive and a quantitative means of detecting biochemical changes in cartilage degeneration prior to morphological or clinical changes [10-12]. Previous studies have demonstrated that T1ρ values are elevated in the cartilages of OA patients when compared with corresponding healthy subjects [13,14]. T1ρ values are correlated with the severity of macroscopic cartilage damage and histologically-assessed PG content in the cartilages of patients with OA or rheumatoid arthritis [15]. Therefore, we used T1ρ mapping to detect early cartilage degeneration associated with degenerative meniscal tears.

The purpose of our study was (1) to detect occult cartilage degeneration in patients with degenerative meniscal tears using T1ρ MRI mapping, (2) to investigate the distribution of T1ρ values in the superficial and deep layers of adjacent femoral and tibial cartilages and (3) to characterise the relationship between early cartilage degeneration and degenerative meniscal tears.

## Methods

### Subjects

This study was granted Institutional Review Board approval at Kyushu University and complied with the Ethical Committee Standards (approval number: 23–75). All subjects provided written informed consent prior to the study. The subjects were recruited from the patients who visited our hospital due to knee joint symptoms. Table 1 shows the baseline characteristics of the subjects included in the study. Anthropometric measurements, including height and weight, were collected, and body mass index (BMI) was calculated for all participants. MRI and radiographic examination was performed on all subjects. Meniscal lesions were graded based on MRI images using the following grading system: grade 0, normal meniscus; grade 1, increased signal intensity of the meniscus without evidence of a tear; grade 2, small radial meniscal tear; grade 3, non-displaced single meniscal tear; grade 4, non-displaced complex meniscal tear; grade 5, meniscal tear with displaced component; and grade 6, macerated meniscal tear [16]. Patients with meniscal changes scoring 2–6 on MRI were assigned to the meniscal tear group. Femoro-tibial angle (FTA) values were calculated based on radiographs, and radiographic severity of OA was determined according to the Kellgren and Lawrence (KL) grading system. The meniscal tear group comprised 22 subjects: Fifteen males and seven females ranging in age from 32 to 73 years (mean, 56.4 ± 12.8 years) with KL grades between 0 and 2. Only patients with frequent symptoms, defined as pain in or around the knee on most days for at least 1 month over the past 12 months and a positive McMurray test, were investigated. Subjects who had severe OA or KL grades more than 3 were excluded, as were subjects with a ligament injury and/or cartilage injury on MRI. To categorise the severity of OA, subjects in the meniscal tear group were further divided into two subgroups: ‘KL normal’ with a KL grade of 0 or 1 (n = 12) and ‘KL mild OA’ with a KL grade of 2 (n = 10). The meniscal tear group consisted of 20 medial and two lateral meniscal tears. The control group comprised 19 healthy individuals, all males and ranging in age from 28 to 54 years (mean, 39.0 ± 7.2 years) without any clinical symptoms of OA or other knee injuries. All controls were only enrolled in the study if they had a KL grade of 0, indicating no signs of radiographic OA.

**Table 1:** **Baseline characteristics and radiographic parameters of meniscal tear group and control group**

	**Meniscal tear group**	**Control group**	***P*** **value**
Characteristic			
Participants, no	22	19	
Age, mean ± SD, years	57.0 ± 14.1	39.0 ± 7.2	< 0.05
BMI, mean ± SD, kg/m^2^	24.4 ± 2.6	23.9 ± 2.1	0.31
Radiographic parameter			
FTA, mean ± SD, °	177.0 ± 2.6	176.7 ± 0.8	0.64
KL grade, no			
Grade 0	4	16	
Grade1	8	3	
Grade2	10	0	

BMI body mass index, FTA femoro-tibial angle, KL Kellgren and Lawrence.

### Magnetic resonance imaging protocols

MRI was performed on a 3-Tesla system (Achieva 3.0 T, Quasar Dual, Philips Healthcare, Best, the Netherlands) using an 8-channel phased-array knee coil. Sagittal fat-suppression turbo spin echo T2-weighted images (FS-T2WI) were obtained using the following parameters: Repetition time/echo time (TR/TE) = 4,675/71 ms, field of view (FOV) = 140 × 140 mm, matrix = 400 × 400, slice thickness = 3 mm, slice gap = 0 mm, flip angle = 90°, bandwidth = 31.54 Hz/pixel, number of slices = 26 and total scan time = 3 min 33 s. FS-T2WI was used as an anatomical reference.

Two-dimensional (2D)-Sagittal T1ρ mapping was calculated from T1ρ-prepared images using the fast field echo technique. The imaging parameters were as follows: TR/T = 4.7/2.4 ms, FOV = 140 × 140 mm, matrix = 320 × 320, slice thickness = 3 mm, slice gap = 0 mm, flip angle = 35°, bandwidth = 31.54 Hz/pixel, spin-lock pulses = 20/1/40/60/80 ms, spin-lock pulse frequency = 500 Hz, number of slices = 26 and total scan time = 16 min 15 s. We used a low flip angle, but it did not affect T1ρ contrast, since we used 6,000 ms of shot intervals between each slice acquisition and filled the k-space using low-high ordering. T1ρ mapping was produced with Philips Research Integrated Development Environment (PRIDE) software written in Interactive Data Language (IDL 6.3, ITT Inc. Boulder, CO, USA) and was used in the quantitative assessment.

### Imaging assessment of T1ρ maps

**Figure 1:**
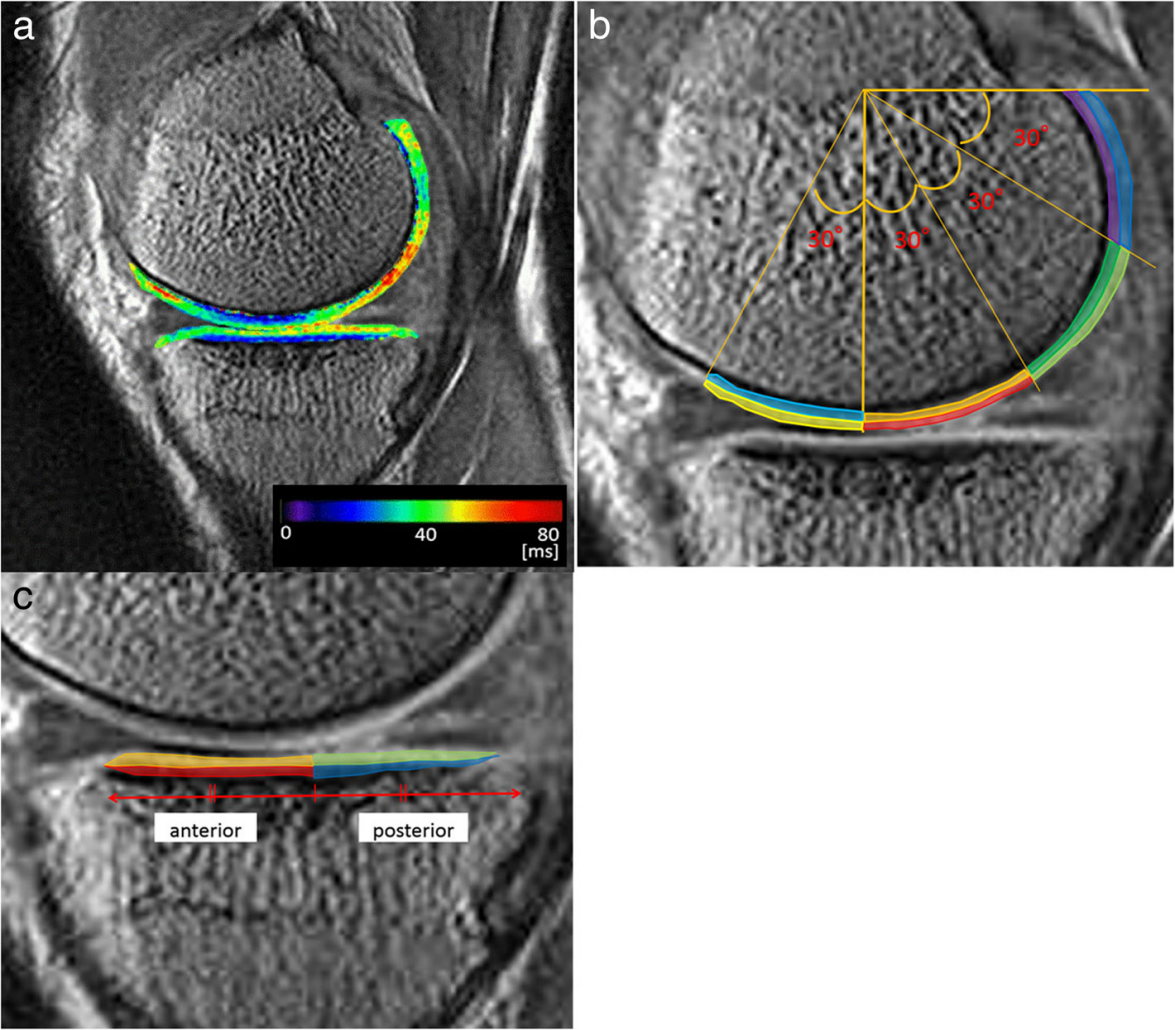
**Assessment of T1ρ values in the articular cartilage. a**. Colored T1ρ mappings of femoral and tibial articular cartilage. **b**. Regions of interest (ROIs) were divided into eight regions on the femoral articular cartilage; superficial (−30°–0°): yellow; deep (−30°–0°): cyan; superficial (0°–30°): red; deep (0°–30°): orange; superficial (30°–60°): yellow-green; deep (30°–60°): green; superficial (60°–90°): blue; deep (60°–90°): purple. **c**. ROIs were divided into four regions on the tibial cartilage; superficial (anterior): orange; deep (anterior): red; superficial (posterior): yellow-green; deep (posterior): blue.

Mean T1ρ values and standard deviations were calculated by two orthopaedic surgeons (H.M. and K.O., with nine and eight years’ experience, respectively) using the ‘Medical Image Processing, Analysis, and Visualization’ software (MIPAV, Biomedical Imaging Research Services Section, Center for Information Technology, National Institutes of Health, Bethesda, MD, USA) (Figure 1a). On the femoral side, regions of interest (ROIs) in the articular cartilage were divided into anterior and posterior parts. The posterior part was further divided into three 30° wedges since the medial femoro-tibial contact point shifts posteriorly until 60° of flexion [17,18], which increases the risk of meniscal injury more than in extended positions. Furthermore, we distinguished superficial and deep layers of equal thickness in all areas (Figure 1b). ROIs of the articular cartilage on the tibial side were divided into anterior and posterior areas, and superficial and deep layers were analysed separately (Figure 1c). In addition, anterior and posterior horn of the meniscus on the same MRI slice was also analyzed. Three adjacent slices at the centre of the medial or lateral compartment in the sagittal plane, respectively, were analysed for each subject. After analysing the ROIs for each of the 14 areas (femoral side, eight areas; tibial side, four areas; meniscus, two areas) on each of the three slices, mean T1ρ values and standard deviations were used for the statistical analyses.

### Statistical analyses

All data are expressed as the mean ± SD. A Mann–Whitney U test was performed to compare T1ρ values for each area on the femoral and tibial articular cartilage between the meniscal tear group and the control group and between genders. Analysis of variance (ANOVA) and a post hoc comparison using Tukey’s honest significant difference test were used to assess differences in T1ρ values for each area on the femoral and tibial articular cartilage between the control, the KL normal and KL mild OA groups. In addition, Pearson’s correlation analysis was applied to study the relationship between patient age and T1ρ values for each area on the femoral and tibial articular cartilage of both the meniscal tear and the control groups. The intra-class correlation coefficient (ICC) was used to describe inter-observer agreement for the measurement of the T1ρ values. ICCs close to 1.0 indicated good agreement between the two observers. All statistical tests were performed with the JMP software version 9.0 (SAS Institute, Cary, NC, USA). A P-value <0.05 was considered statistically significant for each two-tailed analysis.

## Results

**Figure 2:**
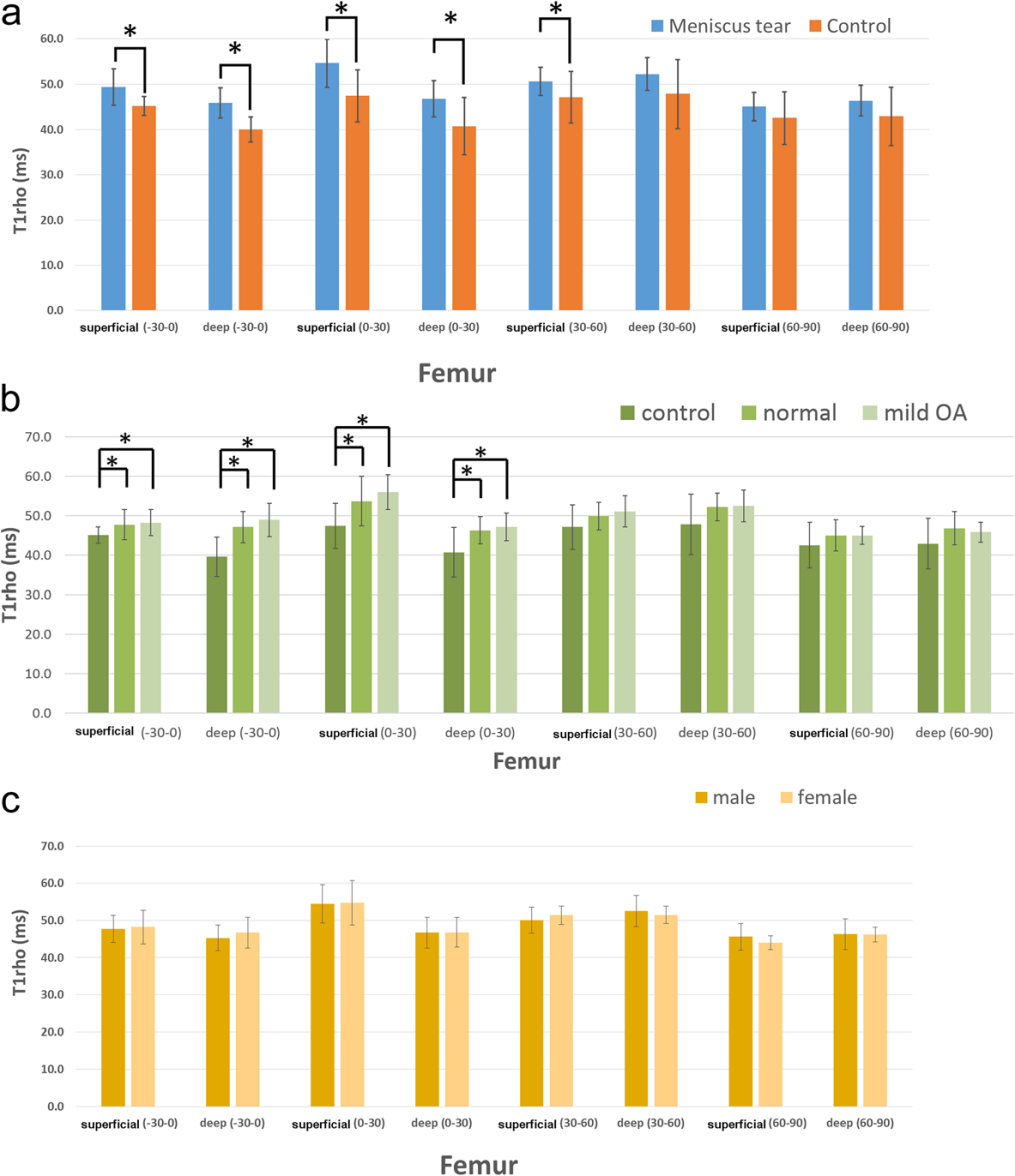
**T1ρ values in different cartilage regions of the femoral condyle. a**. Meniscal tear and control groups **b**. Normal and mild OA **c**. Males and females. Error bars: 95% confidence interval *: P < 0.05.

T1ρ values in each area of the articular cartilage on the femoral condyle and tibial plateau are summarized in Table 2 and Table 3, respectively. For the femoral condyle, T1ρ values in the meniscal tear group were significantly higher than those in the control group in both the superficial and deep regions of the −30°–0° wedge and the 0°–30° wedge, and in the superficial region of the 30°–60° wedge (Table 2 and Figure 2a). With regard to the differences in OA severity between the control group and the two subgroups, a statistically significant difference was observed in both the superficial and deep regions of the −30°–0° wedge and the 0°–30° wedge between the control group and the KL normal group and between the control group and the KL mild OA group. However, no significant differences were observed between the two subgroups in the meniscal tear group (Table 2 and Figure 2b). No statistically significant differences were observed between the genders as well (Table 2 and Figure 2c).

**Table 2:** **T1ρ values (ms) for articular cartilage on the femoral condyle**

	**Superficial (−30°-0°)**	**Deep (−30°-0°)**	**Superficial (0°-30°)**	**Deep (0°-30°)**	**Superficial (30°-60°)**	**Deep (30°-60°)**	**Superficial (60°-90°)**	**Deep (60°-90°)**
Meniscal tear group	49.0 ± 4.0	45.2 ± 3.3	54.6 ± 5.3	46.8 ± 4.0	50.5 ± 3.1	52.2 ± 3.6	45.4 ± 3.1	46.2 ± 3.4
Control group	45.1 ± 2.1	39.6 ± 5.0	47.4 ± 5.7	40.7 ± 6.3	47.1 ± 5.7	47.8 ± 7.6	42.5 ± 5.8	42.9 ± 6.4
P-value	<0.05	<0.05	<0.05	<0.05	<0.05	0.08	0.29	0.15
Comparison between the OA severity groups								
Normal (n = 12)	47.7 ± 3.8	47.1 ± 4.0	53.2 ± 6.3	46.1 ± 4.2	49.8 ± 3.5	51.9 ± 3.5	45.0 ± 4.0	46.3 ± 4.2
Mild OA (n = 10)	48.2 ± 3.3	48.9 ± 4.2	56.0 ± 4.4	47.1 ± 4.0	51.1 ± 3.5	52.5 ± 4.0	45.0 ± 2.3	45.8 ± 2.5
P-value	0.88	0.72	0.18	0.87	0.62	0.84	0.87	0.71
Comparison between genders								
Male (n = 15)	47.7 ± 3.7	45.3 ± 3.5	54.6 ± 5.2	46.9 ± 4.1	50.1 ± 3.5	52.6 ± 4.2	46.0 ± 3.6	46.3 ± 4.1
Female (n = 7)	48.2 ± 4.5	46.7 ± 4.1	54.7 ± 6.0	46.8 ± 4.0	51.4 ± 2.5	51.5 ± 2.4	44.0 ± 1.9	46.2 ± 2.0
P-value	0.25	0.32	0.33	0.80	0.29	0.53	0.32	1.00

**Table 3:** **T1ρ values (ms) for articular cartilage on the tibial plateau**

	**Superficial (anterior)**	**Deep (anterior)**	**Superficial (posterior)**	**Deep (posterior)**
Meniscal tear group	52.0 ± 4.3	41.3 ± 3.7	53.1 ± 5.1	42.8 ± 4.3
Control group	46.7 ± 5.4	39.6 ± 5.0	46.0 ± 4.9	41.3 ± 4.8
P-value	<0.05	0.28	<0.05	0.55
Comparison between the OA severity groups				
Normal (n = 12)	51.7 ± 4.5	41.6 ± 3.9	52.9 ± 5.5	43.0 ± 4.9
Mild OA (n = 10)	51.8 ± 4.3	40.4 ± 3.3	52.3 ± 4.8	41.6 ± 3.6
P-value	1.00	0.49	0.37	0.22
Comparison between genders				
Male (n = 15)	52.5 ± 5.0	41.6 ± 4.3	55.0 ± 4.7	42.5 ± 4.7
Female (n = 7)	50.6 ± 2.4	40.6 ± 2.3	51.0 ± 5.3	42.4 ± 4.1
P-value	0.70	0.64	0.19	0.97

**Figure 3:**
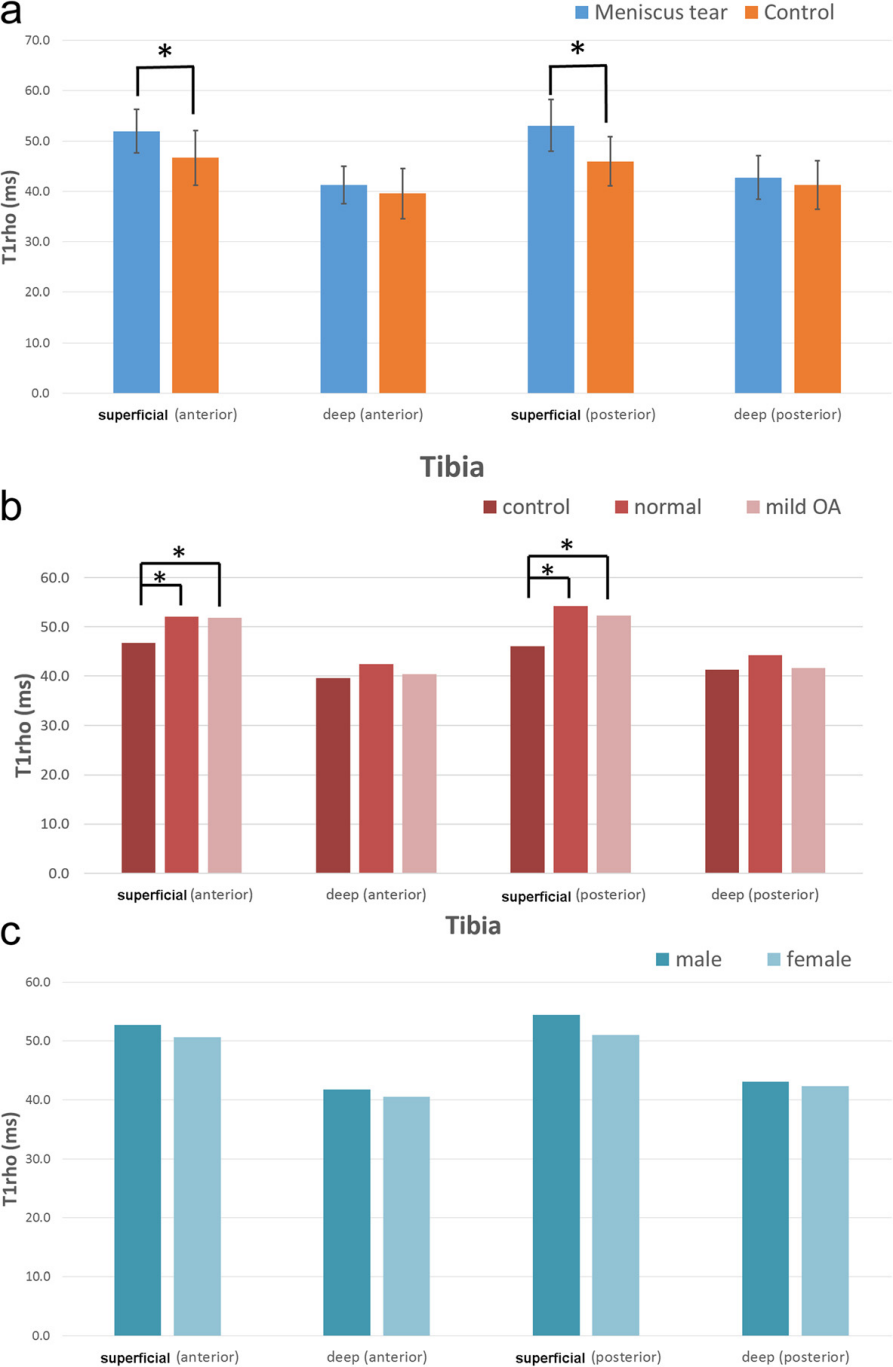
**T1ρ values in different cartilage regions of the tibial plateau. a**. Meniscal tear and control groups. **b**. Normal and mild OA. **c**. Males and females. Error bars: 95% confidence interval *: P < 0.05.

For the tibial plateau, the meniscal tear group had significantly higher T1ρ values than the control group in both the anterior and posterior regions in the superficial areas (Table 3 and Figure 3a). With regard to the differences between the control group and the two subgroups in the meniscal tear group, a statistically significant difference was observed in the superficial areas of both the anterior and posterior regions between the control group and the KL normal group, and between the control group and the KL mild OA group. However, no statistically significant differences were observed between the two subgroups in the meniscal tear group or between the genders, similarly to the findings related to the femoral condyle (Table 3 and Figure 3b and c). The T1ρ values (ms) of the posterior horn of meniscus were significantly higher in the meniscal tear group than those in the control group (44.2 ± 3.5 vs 35.6 ± 2.1, respectively, p < 0.01). There were no significant differences in T1ρ values of the anterior horn of meniscus between in the meniscal tear group and the control group (36.0 ± 2.1 vs 34.4 ± 3.1, respectively).

**Figure 4:**
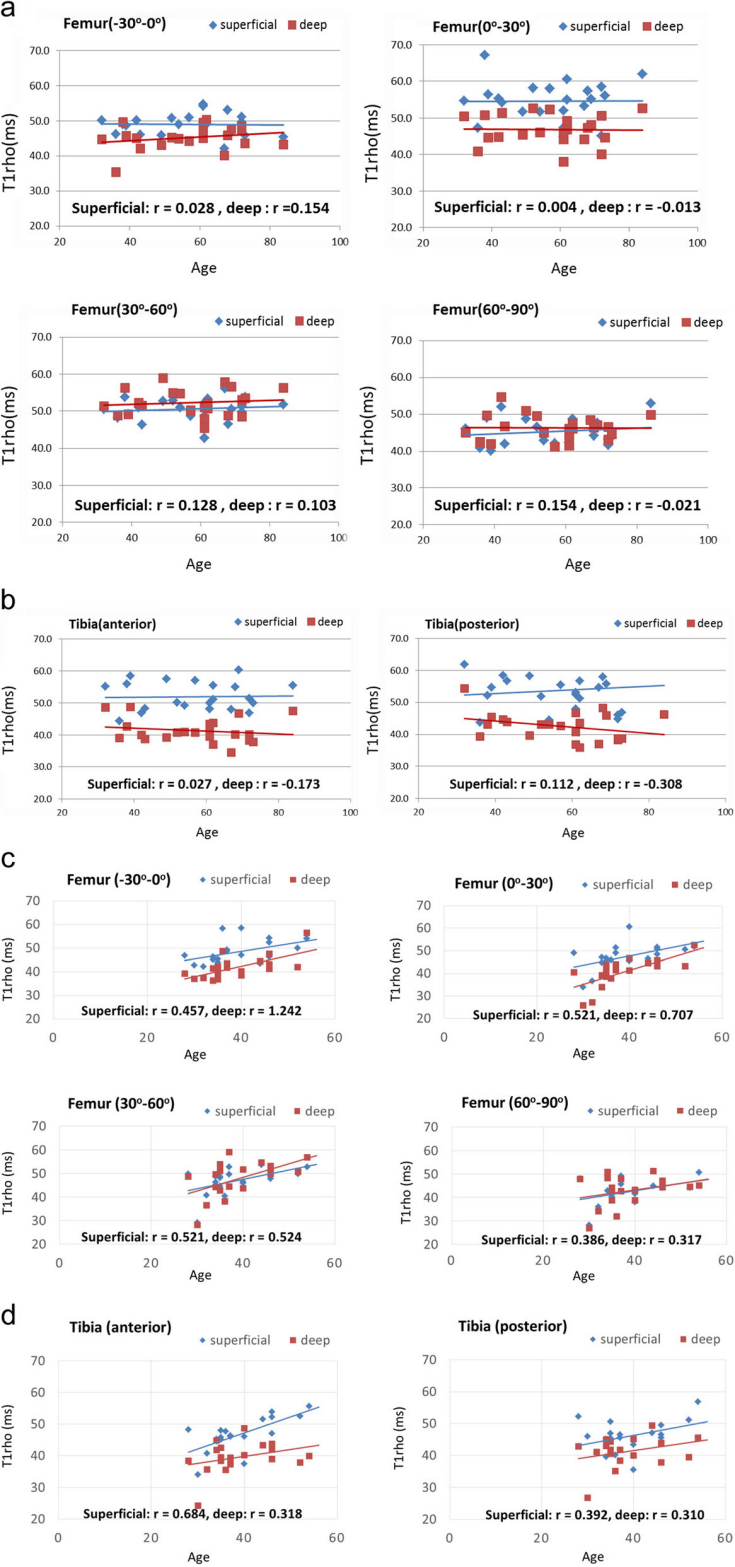
**The relationship between age and T1ρ values of different cartilage regions. a**. Femoral condyle of the meniscal tear group. **b**. Tibial plateau of the meniscal tear group. **c**. Femoral condyle of the control group. **d**. Tibial plateau of the control group.

No significant correlations were observed between age and T1ρ values at any regions of both the femoral condyle and the tibial plateau in the meniscal tear group (Figure 4a and b), but slight or moderate correlations were observed between these in the control group (Figure 4c and d). The intra-class correlation coefficient (ICCs) of the T1ρ values between the two observers was 0.93 (95% CI = 0.84–0.95), indicating good agreement between the observers.

## Discussion

In the present study, we evaluated sub-regional and layer-specific T1ρ values of femoro-tibial articular cartilage in patients with meniscal tears with no or mild radiographic OA and compared them with those of healthy subjects. Higher T1ρ values were observed at the distal area of the femoral condyle and the tibial plateau in the meniscal tear group, even in the subgroup with no radiographic OA. Hence, the study suggested the association between meniscal tears and occult cartilage damage.

However, this study had several limitations. First, the mean age was different between the meniscal tear group and the control group, and the average age of the subjects in the meniscal tear group was relatively high. It was challenging to recruit age-matched control subjects from volunteers. In fact, there were weak or moderate correlations between age and T1ρ values in articular cartilage in the control group. However, no correlations were observed between age and T1ρ values in the meniscal tear group. Patients less than 50 years old with meniscal tears also showed relatively high T1ρ values. Therefore, the increased T1ρ values in the meniscal tear group were not attributed to the relatively higher age of this group. Second, the magic angle effect may affect the calculation of T1ρ values in articular cartilage, especially in the 30°–60° area. This area is susceptible to the magic angle effect both superficially and deeply since this effect increases T1ρ values relating to the orientation of collagen fibrils in articular cartilage and a static magnetic field [19,20]. Therefore, attention should be paid to results showing that T1ρ values are significantly higher in this area, when comparing different areas in the same knee. However, this study compared the same area between groups of subjects, indicating that T1ρ values in both superficial and deep regions of the 30°–60° area in the meniscal tear group were significantly higher than those of the control group. Therefore, there is no doubt that cartilage denaturation had occurred in association with a degenerative meniscal tear in these areas. Third, ROIs were manually drawn around the articular cartilage boundaries to calculate T1ρ values. Factors that might cause miscalculation include the misidentification of articular cartilage boundaries, such as that between the superficial and deep layers. However, ICCs indicated good agreement between the observers, and so any accidental error associated with drawing the ROIs would not significantly affect the conclusions of this study. Finally, the relatively small sample size and the cross-sectional design are other limitations of the study. It was also difficult to evaluate other potential confounders influencing the T1ρ values with multivariable analysis.

Previous studies have evaluated the relationship between cartilage change and OA using a variety of methods. Bassiouni et al. examined the cartilage in OA using phonoarthrography, musculoskeletal ultrasonography and biochemical biomarkers and concluded that phonoarthrography and musculoskeletal ultrasonography could be used as parameters for following up cartilage disorders in OA knees [21]. T1ρ-weighted MRI methods have been proposed recently as an attractive potential biomarker to evaluate biochemical changes in the cartilage matrix non-invasively [10,11]. It has been reported that T1ρ relaxation time is sensitive to early biochemical changes in cartilage, especially the PG content [12,15]. In the early stages of OA, PG depletion occurs prior to the minor structural changes of collagen (denatured collagen) [15]. T1ρ MRI mapping is a useful imaging method to detect this PG depletion, and minor changes of denatured collagen may also contribute to the T1ρ signal. Takayama et al. reported that T1ρ mapping is superior to T2 mapping for the assessment of denatured articular cartilage in the early stages of OA, with a capability to assess the severity of cartilage degeneration with good accuracy [22]. This study utilized T1ρ MRI mapping to detect early cartilage damage in patients with degenerative meniscal tears and healthy subjects.

Some investigators have compared T1ρ values of sub-regions and the whole area of the femoro-tibial cartilage and menisci in patients with OA [23], whereas others investigated the relationship between T2 relaxation values within the superficial zone of the articular cartilage following different types of meniscal degeneration/tear [24]. However, most of these previous studies assessed a limited or region-specific area of the entire joint, such as in one slice of the mid-sagittal plane of the medial and lateral femoral condyles. Although it is known that the severity of degeneration is heterogeneous and focal within the OA joint, there is still a lack of information about the regions of the weight-bearing area in the femoral condyles and the tibia plateaus that are likely to be affected by the early degenerative changes associated with a meniscal tear. Furthermore, superficial and deep layer-specific assessment of the articular cartilage might be more sensitive in terms of detecting early changes in the cartilage macromolecular structures. In the present study, femoral articular cartilage in the meniscal tear group had significantly greater T1ρ values at the distal area, both superficially (−30°–0°, 0°–30° and 30°–60° areas) and in the deep regions (−30°–0° and 0°–30° areas). In tibial articular cartilage, significantly higher T1ρ values were found superficially in both anterior and posterior areas in the meniscal tear group than in the control group. In both femoral and tibial articular cartilage, no significant differences in T1ρ values between the two subgroups of OA severity in the meniscal tear group were observed. This finding suggests that degenerative meniscal tears are associated with occult cartilage damage in the very early stages of OA rather than radiographic OA severity, and supports the previous studies that have reported the direct interaction between meniscal and cartilaginous abnormality [1,8,23,24]. Hence, occult cartilage damage exists in patients with a symptomatic meniscus tear even though there is no evidence of radiographic OA. The meniscus plays an important role in maintaining the integrity of the articular cartilage by reducing the contact impact forces between the articular surfaces. Damage to the meniscus increases the contact peak stress on the femoral and tibial articular cartilage surfaces and alters the biomechanical loading of the joint, putting the patient at increased risk of cartilage degeneration [18,25].

Previous studies using T1ρ or T2 MRI mapping have demonstrated a relationship between meniscal and cartilage morphology as well as the cartilage biochemical composition [23,24]. Wang et al. demonstrated that regional damage of both femorotibial cartilage and menisci was associated with the severity of OA using T1ρ MRI mapping in subjects with various stages of OA [23]. They reported that the T1ρ values of femoral anterior cartilage sub-region and the medial posterior sub-region of the meniscus are both higher in moderate-severe OA than those in doubtful-mild OA. However, it was unclear whether symptomatic meniscal tear was associated with occult cartilage damage in patients without OA. Kai et al. established an association between meniscal signal-complex tears and increased T2 values in tibial articular cartilage [24]. They reported that T2 values of femoral condyle cartilage was not associated with meniscal tear. The differences between T1ρ and T2 in the sensitivity and diagnostic power for the detection of early macromolecular changes in cartilage might have caused the discrepancy between their results and ours. Furthermore, research into the investigation of the heterogeneity of cartilage T1ρ values in relation to joint morphology is still new. This study suggests that a heterogeneous distribution of cartilage denaturisation associated with meniscal degenerative tears is a precursor of future OA progression.

Additionally, the facts obtained in this study suggest a potential risk for progression of cartilage damage after a meniscal surgery for these patients despite no radiographic OA being observed. Clinical application of this MRI technique as a routine evaluation for meniscal tear could be a help for surgeons. A longitudinal study should be able to reveal whether preoperative evaluation of cartilage macromolecular quality using this MRI technique can predict postoperative progression of cartilage damage.

## Conclusions

This study suggested that degeneration of the cartilage matrix had already occurred in patients with degenerative meniscal tears although morphological changes of the articular cartilage had not been detected. This change appeared in the superficial zone of the articular cartilage at the point where femoral and tibial cartilage contact each other during slight flexion. Our results suggest that this area is the origin of early biochemical changes leading to OA progression associated with degenerative meniscal tears.

## Abbreviations

OA: Osteoarthritis
MRI: Magnetic resonance imaging
PG: Proteoglycan
KL: Kellgren-Lawrence
ICC: Interclass correlation coefficient
ROI: Region of interests
